# Predictive genetic testing in amyotrophic lateral sclerosis (ALS): Experiences of decision‐making and engagement with UK genetic counseling services

**DOI:** 10.1002/jgc4.70184

**Published:** 2026-02-19

**Authors:** Jade Howard, Hilary L. Bekker, Christopher J. McDermott, Alisdair McNeill

**Affiliations:** ^1^ Division of Neuroscience, Sheffield Institute for Translational Neuroscience The University of Sheffield Sheffield UK; ^2^ Leeds Unit of Complex Intervention Development (LUCID), Leeds Institute of Health Sciences, School of Medicine University of Leeds Leeds UK; ^3^ Academic Directorate of Neuroscience Royal Hallamshire Hospital Sheffield UK; ^4^ Sheffield Clinical Genetics Service Sheffield UK

**Keywords:** amyotrophic lateral sclerosis, decision‐making, information needs, motor neuron disease, predictive genetic testing, qualitative research

## Abstract

Predictive genetic testing enables at‐risk relatives of people with amyotrophic lateral sclerosis (ALS, also known as motor neuron disease or MND) to find out if they have inherited the genetic variant identified in their family member and have an increased chance of developing symptoms. As research progresses, eligibility for and interest in predictive testing is increasing. This paper explores the experiences of people making decisions about predictive testing and identifies their information needs over the process. Semi‐structured interviews were carried out with 14 individuals from across the United Kingdom who had, or were considering, predictive testing for ALS. Interviews were carried out via video call or face‐to‐face between March and September 2023, transcribed, and analyzed using inductive framework analysis. Findings illustrate a range of experiences. Interviews suggest variation in practice in terms of the structure of the genetic counseling process and the content of information given. Some expressed positive experiences of genetic counseling, and valued feeling listened to, understood, and supported. Others perceived barriers to accessing testing, felt the information provided was directive or not sufficient to support their concerns and decision‐making. Information needs varied, and whilst people felt satisfied, there were also diverse, unmet information and support needs raised throughout the decision‐making process and beyond. This evidence has been used to support the development of a patient decision aid for predictive genetic testing in ALS.

**What is known about this topic**

Predictive testing in ALS is a complex and personal decision. Whilst genetic counseling can support the decision process, little is known about how individuals proceed through the genetic counseling and testing pathway, and what information people need to support their decisions and understand the wider personal and family implications.

**What this paper adds to the topic**

This paper explores experiences of making decisions around predictive testing and of the genetic counseling and predictive testing process. It highlights areas of supportive practice, as well as areas where people had unmet needs and unanswered questions. This evidence has informed a decision aid to proactively support people making decisions about predictive testing.

## 1 INTRODUCTION

Amyotrophic lateral sclerosis (ALS, also known as motor neuron disease or MND) is a neurodegenerative condition that leads to muscle weakness and wasting. Clinical symptoms vary but include difficulties with speech, movement and breathing (Brown & Al‐Chalabi, 2017). In around 10–20% of individuals, the condition is associated with a pathogenic monogenic variant in one of around 40 identified genes (Akçimen et al., 2023; Goutman et al., 2022; Shepheard et al., 2021). A genetic cause can now be identified in up to 75% of people with a family history, as well as a smaller number of individuals with no known family history (Akçimen et al., 2023; Chia et al., 2018). First degree relatives of individuals with an identified pathogenic variant can choose to have predictive genetic testing, sometimes called pre‐symptomatic testing, to find out whether or not they have inherited this gene variant (Roggenbuck et al., 2017). ALS‐linked gene variants are commonly associated with autosomal dominant inheritance, meaning children of those with an identified gene variant have a 50% inheritance risk (Akçimen et al., 2023; Goutman et al., 2022). Reproductive genetic testing options may be available where there is an identified ALS‐linked gene variant in the family (Dharmadasa et al., 2022).

In the United Kingdom, ALS predictive genetic testing is organized within clinical genetics services. Clinical guidelines follow a similar model of care used to support predictive testing in Huntington's disease (Crook et al., 2017; Roggenbuck et al., 2017), where genetic counseling is advocated to reduce the potential risk of harm that can result from testing (Dharmadasa et al., 2022). It is recommended that individuals have at least two genetic counseling consultations prior to making a decision about giving a blood sample for testing, with a time gap in between. For people who choose to have testing, a further consultation is advised to receive and discuss the test results (Crook et al., 2017; Dharmadasa et al., 2022).

Genetic counseling is a specialist type of person‐centered healthcare communication involving the provision of balanced and non‐judgmental information. It is aimed at supporting individuals to understand their disease risk and make voluntary and informed decisions that align with their values, life, and family circumstances (Jamal et al., 2020; Resta et al., 2006). Central to these conversations is providing a structure to help people to explore what might change in their life in light of their predictive testing decision, and the consequences of having or not having testing (Jamal et al., 2020). In this complex area, where there is currently limited clinical intervention to modify the disease (e.g., no approved preventative options beyond reproductive genetic testing), and where the perceived benefits and drawbacks of testing are grounded in personal priorities and values, enabling patient autonomy and informed decision‐making is key.

The distinct characteristics of ALS, its treatment landscape, and genetic complexities, have prompted calls for a disease‐specific guideline for predictive testing (McNeill et al., 2022). Counseling individuals on the implications of predictive testing is complicated by the clinical heterogeneity of ALS‐linked gene variants and evolving knowledge of ALS genetics (Al‐Chalabi et al., 2017; Crook et al., 2017). Gene variants usually show incomplete penetrance, meaning people testing positive may never develop symptoms. Further, it is not possible to predict when symptoms may develop (Al‐Chalabi et al., 2017; Dharmadasa et al., 2022). In some gene variants, other neurological symptoms are associated, for example, the *C9orf72* repeat expansion, the most common genetic cause of ALS in a white European population, can also cause frontotemporal dementia (FTD) or a mixed phenotype (DeJesus‐Hernandez et al., 2011). Particular gene variants may be associated with patterns in penetrance, presenting phenotype, and disease progression—though these factors can be vastly different within the same family (Dharmadasa et al., 2022; Shatunov & Al‐Chalabi, 2021).

Until recently, there have been no disease modifying treatments for people with ALS, and survival is on average 2–4 years from diagnosis (Akçimen et al., 2023; Goutman et al., 2022). However, this is a fast‐moving landscape, and clinical trials are underway internationally, investigating the utility of genetically targeted treatments and their impact on symptoms and disease progression. Notably, Tofersen has been approved in the United States and elsewhere as a treatment of *SOD1* ALS, with approval pending in the United Kingdom (Biogen, 2023). A pre‐symptomatic trial is underway to establish if this can delay or prevent symptoms in gene carriers, with implications for the uptake of predictive testing and pressures on genetic counseling services (Crook et al., 2022; De Oliveira et al., 2023; Fontaine et al., 2024).

Research suggests decision‐making around predictive testing in ALS is a varied process, and can be challenging for relatives (Howard, Forrest Keenan, et al., 2024; Fanos et al., 2011). We know from previous research that people in the United Kingdom have limited and inconsistent access to dedicated resources when making ALS predictive testing decisions and going through the process (Howard et al., 2023). Making this decision well may be confounded by gaps in the information provided, access to services, knowledge about the ALS trajectory and treatment options, changing personal goals and values, involvement of family, and the dynamic field of technological advances around genetic testing and targeted therapy. This study aims to explore participants' experiences of making decisions about predictive testing and their experiences and support needs when engaging with genetic counseling services.

## 2 METHODS

### 2.1 Context

This study is part of a project to develop patient decision aids to support people with ALS and their family members to make informed decisions about genetic testing. Patient decision aids are evidence‐based resources that guide people to make decisions between healthcare options, based on their personal values and priorities (Stacey et al., 2024). Patient decision aids are informed by evidence of how people make healthcare decisions, and how they are supported by services in making their decision along the pathways of care (Bekker, 2010; Bekker et al., 2003). This study adds to the evidence base from our studies investigating (a) resources used by services to support people when making predictive and genetic testing decisions (Howard, Bekker, et al., 2024, 2023); (b) UK health professionals' training needs in providing genetic testing as part of ALS care (Howard, Bekker, et al., 2024); and (c) the needs and preferences of people living with ALS making genetic testing decisions (Howard, Bekker et al., 2025; Howard, McNeill et al., 2025a, 2025b). Development of the decision aids has been guided by Bekker's multiple decision‐makers framework 'Making Informed Decisions Individually and Together (MIND‐IT) in healthcare' (Bekker et al., 2023), the Medical Research Council research framework for developing and evaluating complex interventions (Skivington et al., 2021), and the International Patient Decision Aid Standards collaboration (IPDAS) criteria (Joseph‐Williams et al., 2014). The research and outputs of this project have been supported by a steering committee comprising clinicians, researchers, and people with lived experience of ALS. The committee offered feedback on the study design and methods, analysis, and implications of findings for the intervention development phase of the project. The decision aids (“Genetic testing and MND,” and “Predictive genetic testing and MND”) are now publicly available (https://mymndgenetest.shef.ac.uk/).

### 2.2 Design

This study employed qualitative methods to explore participant views about the predictive testing decision and its consequences. Semi‐structured interviews were guided by a topic guide, which was used flexibly (see Data S1). The topic guide was developed from relevant clinical and decision support literature, and the experience of the study team. With backgrounds in psychology, social science and health services research, clinical genetics, and neurology, the team brought varied clinical and methodological expertise to the project. The topic guide was structured around the following areas: experiences of ALS in the family; understanding ALS genetics and finding out about inheritance risk; experiences of predictive testing; and information and support needs.

Ethics approval for this research was granted by a UK NHS Research Ethics Committee (22/SW/0047) and the University of Sheffield (050846).

### 2.3 Participants

Interviews were carried out between March and September 2023 with 14 participants who had, or had considered, predictive testing for ALS‐linked gene variants. Participants were recruited through a UK NHS clinical genetics center and the MND Association (an equal number from each). For the former, they were approached by the clinical team and given a recruitment pack including a participant information sheet; for the latter, the recruitment pack was sent when they contacted the research team directly.

Recruitment was stopped when interviews were deemed to hold sufficient “information power” (Malterud et al., 2016). Information power is based on the principle that the more relevant information the sample holds, the fewer participants are needed. In this research, key aspects of the sample considered by the authors in determining information power were the clearly defined research questions (on experiences of genetic counseling/ predictive testing and information and support needs); the purposive sampling method (which guided recruitment of participants who had had, and not had, predictive genetic testing); and the rich and focused narratives shared in interviews.

### 2.4 Procedure

At the point of expressing interest in the study, each participant was contacted by phone or email to arrange the interview and consent process; all were given the chance to ask questions. Participants were asked briefly to describe their experience of ALS and predictive testing if they had not offered this information to confirm eligibility for the study. Demographic details were noted where offered, with participants asked to clarify any outstanding details not previously given at the interview itself.

In‐depth, semi‐structured interviews were carried out by JH, a female researcher with experience of conducting interviews with families affected by inherited forms of ALS. Participants were interviewed individually and took part in a single interview. Interviews were held virtually via Google Meet or face‐to‐face in a hospital meeting room, at the preference of the participant. In the latter case, travel costs were reimbursed.

Interviews lasted an average of 1 h 10 min and were audio recorded for transcription purposes. Interviews were transcribed verbatim by professional transcribers. Transcripts were not returned to participants. Participants were asked if they'd like to be kept up to date with the study.

### 2.5 Analysis

Analysis was carried out alongside interviews by JH. An inductive framework analysis approach was used (Ritchie et al., 2014), facilitated by NVivo software. The analysis steps involved: familiarization with the data through word‐for‐word transcript checking and repeated reading; inductive coding; development of an initial thematic map, using a constant comparison approach; indexing and sorting data (and refinement of the thematic map); development of a framework to display the data for each subtheme. Frameworks were then reviewed and developed, with key elements and dimensions identified to explore the breadth of experiences. The developing analysis was discussed regularly within the multidisciplinary study team (described above). A reflexive diary was kept by JH of queries, points of interest, and the evolution of the analysis throughout the process.

Codes focusing on predictive testing and information and support needs were developed into the following themes: understanding the decision and consequences of predictive testing; accessing predictive testing and the genetic counseling process; navigating decision‐making and genetic counseling support; making sense of ALS and being at risk; and satisfaction with genetic counseling and information provision.

## 3 RESULTS

Participants included eight individuals who had had predictive testing, with six testing positive, one testing negative, and one individual waiting for results. Six had not had a predictive test at the time of the interview; these individuals were at various stages of decision‐making and some were at different points in the genetic counseling pathway (see Table 1 for participant characteristics).

**TABLE 1:** Participant characteristics.

Participant characteristics	Number of participants (*n* = 14)
Age	
20–29	1
30–39	4
40–49	3
50–59	4
60–69	2
Gender	
Woman	6
Man	8
Ethnicity	
White British	12
Indian	1
Chinese	1
Gene variant in the family	
*C9orf72*	8
*SOD1*	2
*FUS*	1
Does not remember	1
Unknown	2
Predictive genetic testing status	
Tested positive	6
Tested negative	1
Not tested	2
Not tested—in process of genetic counseling	3
Not tested—waiting for genetic counseling referral	1
Not tested—waiting for results	1

Participants were aged from their early 20s to late 60s. Six were women and eight men. The majority identified as white British. Participants were at risk of gene variants *C9orf72*, *SOD1*, and *FUS*. One participant did not remember the identified gene variant in their family. Two did not know because their family members with ALS had not been tested but they identified as “at risk” based on family history. To note, these latter two participants would not be eligible for predictive genetic testing according to current UK guidance, but are eligible for genetic counseling to seek information and discuss their risk. Their perspectives were included as they were engaging with the predictive test decision (described further below) and the authors decided it was important to include participants living with different levels of (un)certainty as to their inheritance risk. The results below present the needs of people who've had predictive testing reflecting back on their past experiences, and people who have not had predictive testing discussing their needs and reasoning as they engage with the decision.

No participants dropped out of the study. Researcher‐assigned pseudonyms have been used throughout. Quotations are accompanied by descriptors to indicate participant age and predictive testing status. Quotations have been lightly edited for readability.

### 3.1 Understanding the decision and consequences of predictive testing

Participants identified the need to understand the purpose and process of predictive testing, and the consequences of having and not having the test, before making their decision. Additionally, they expressed a need for signposting to information and support around the “longer term” decisions and options that can arise after predictive testing.

For people who had not had predictive testing, there was a need for additional details about the options available, the genetic testing process and associated timelines, and what to expect from genetic counseling consultations.

Robert (50–59, not tested) wanted more explanation about the testing process to inform decision‐making: “I think knowing the timeframe from seeing a counsellor, getting the test, and then finding the result would be of benefit to people. So that they can make a more informed decision as to whether they get that test now or they get it further down the line.”

Melanie's father had opted to have his DNA stored after his ALS diagnosis. She was unsure of her own options and the process she would need to go through to pursue predictive testing: “I just have a lot of questions about how to go about it” (Melanie, 40–49, not tested). For Melanie, predictive testing was positioned as an intertwined decision as she thought about whether to have her father's DNA sample tested.

Ben (40–49, not tested) highlighted that being informed of the test accuracy was important for decision‐making: “How often are they wrong?… if it's positive or negative, they should do another couple of tests just to make sure. Because this is potentially life changing information.”

Participants wanted to know the potential ramifications of testing or not testing for themselves and their relatives, including financial implications:

I found out that it doesn't affect my travel insurance, but I had to do a lot of searching … All these things may seem small but they can make all the difference to the decision that you make to be tested. So a flow‐chart of ‘Have you thought of this? Have you thought about the emotions? Your family members, have you thought about what it means for distant relatives?’ (Prakash, 60–69, not tested)

Participants also felt it was important to think through the potential emotional and psychological consequences of a positive result:

If we could somehow capture subjective wellbeing pre and post test and then we could actually inform people, as a general rule, having the test is an improvement or a hinderance. (Toby, 30–39, not tested)

Communication within the family and sharing of genetic risk information, both now and in the future, was also a concern for participants: “the thing that comes to mind is, is once you know, then what do you do with that information?… Do you hold onto it yourself, or are you obliged to tell your kids?” (Robert, 50–59, not tested). Participants hoped genetic counseling would provide a space to talk this through.

Participants who had predictive testing highlighted the need for details not explained sufficiently before they'd had the test and received their results.

Megan had not been informed how her result would be presented, but after testing positive, she received a letter saying she could find out how many “repeats” she had. She had to do her own research to work out what this meant and decide whether to accept or not.

Craig (50–59, tested positive) had expected to receive support following his test result, and felt disappointed about what he was offered. He believed people should be told what post‐test support is available in pre‐test counseling, to inform decision‐making: “The only thing I think missing from that process is being told at that point when you agree to go ahead with the test what help and support you can get if you get a positive test result.” He was one of several participants who highlighted a desire for information on research participation opportunities. Megan (30–39, tested positive) had explored opportunities herself and volunteered for one study: “Nobody told me about that but again, it helped me to feel like I was doing something.”

Family dynamics, and the emotional impact of disclosure following receipt of results, were seen as topics requiring more discussion during genetic counseling consultations: “There are emotions around siblings… I had a thought of, ‘how will I feel if my sister's negative?’ Even having the thought makes you feel like, ‘Am I a bad person?’ because obviously I want her to be negative… but then, it's that, will there be guilt, will there be any animosity?” (Julia, 30–39, tested positive).

Another worry I have is how the hell am I going to tell my kids this when they are old enough?… So I think support around how to tell your kids, what do you tell them, are you sure you want to know about this? (Megan, 30–39, tested positive)

For some participants, predictive testing was intertwined with reproductive choices. Martina (40–49, tested positive) appreciated factual information on pre‐implantation genetic testing, which informed her decision to have predictive testing: “That was never shied away from, that physically it's quite something to go through, emotionally it's quite something to go through… the odds of it happening and being successful are really not that amazing. So I think that's all really important information when you're making that decision.”

Prakash (60–69, not tested) felt more information was needed on reproductive options: “I think definitely they should be educating people more, to say, “Well this is available”… if I knew 25 years ago, I would have really considered it. That would have changed my decision to know.”

### 3.2 Accessing predictive testing and the genetic counseling process

People described varied experiences of the genetic counseling process. In line with current guidelines, most participants were referred to clinical genetics for genetic counseling, which was provided by clinical genetics consultants, genetic counselors, or both. The process generally involved two or more consultations before the test, with a time gap in between. Ben (40–49, not tested) was unsure whether the discussion he'd had constituted genetic counseling: “Not had counselling. Had an interview about whether or not I wanted to have it done.”

People's satisfaction with the consultations varied. Megan (30–39, tested positive) felt her genetic counseling was carried out efficiently: “Obviously, she had a process that she had to go through and she went through it, but she didn't draw it out longer than it needed to be.”

Participants also described frustration at the rigidity of the genetic counseling process, with the clinical guidance perceived as a barrier to receiving personalized care:

The reason that I'd gone for the genetic testing was ‘I'm on mat leave, I've got the time to process this’, and he [clinician] was like, ‘No, go away and wait for three months’. And I was like, ‘But then I'm going to be hitting my time back to work… juggling two kids and the school run’… and he was like, ‘No, three months…’, and I was like, ‘You're not listening, I know I want it, you're not going to change my mind, time is not going to change my mind’ (Julia, 30–39, tested positive)

It was like, ‘Oh, just have some more time to think about it’. It was like, ‘I've had over a year since my Mum's diagnosis to think about it, process it’…I don't think you can put a set time on someone, because I think everybody, like I said, deals with it differently (Tori, 20–29, tested positive)

In addition, participants discussed their relatives' genetic testing decisions, which at times impacted their own experience of genetic counseling. Ben described being asked by his genetics team to encourage his father to consider having a predictive test before he went ahead; if positive, his own result would mean his father also carried the gene variant.

Julia's sister was interested in predictive testing and had attended Julia's consultation. However, she was told she was “out of catchment” and would need a referral through her own GP. Tori felt genetics services should be set up to facilitate genetic counseling as a family unit where requested.

It is worth noting that genetic counseling may not be accessed by some who could benefit, due to perceptions that it is most useful to those who had already decided to go ahead with a predictive test: “I know you can have the counselling and then decide not to, but I just think that that's a waste of resources to me… But there should be some kind of counselling if you're at risk and kind of struggling” (Robert, 50–59, not tested).

### 3.3 Navigating decision‐making and genetic counseling support

Participants often sought out genetic counseling having already decided to have predictive testing. Others sought genetic counseling to learn more about testing and discuss their options before reaching a decision. Even when participants state they have made a decision, one of the aims of genetic counseling is to explore the decision options and their consequences and help people reflect on the impact for themselves and their relatives. Participants had varied reflections on the process:

Angus (30–39, waiting for results) appreciated having the options laid out, including the option to store his blood sample until a later point, or have the test and decline to find out the result: “Having that clarity and practicality of someone just laying it all out really clearly was so useful and so helpful because it really meant that I had some time to reflect”. He valued having the process explained, including what would happen after his result and what support would be available.

Toby (30–39, not tested) valued genetic counseling as an opportunity to talk through his feelings around predictive testing: “It was less about seeking information for me but more about kind of being able to weigh it all up with somebody else.”

Prakash (60–69, not tested) described his consultation as “high level,” more about information provision than exploring emotions: “There was a little bit about feelings but I don't think there was enough of that. So it was more me asking the questions.”

Megan (30–39, tested positive) expressed frustration at the genetic counseling process: “I knew that I'd be ok with it but I felt like I had to convince somebody else of that. You know, that's just a bit frustrating when you just want to get on with it.”

A key purpose of genetic testing is to support the person to make an informed decision that aligns with their values and life. There was variation in participants' perception of the non‐directiveness of pre‐test consultations.

Evelyn (60–69, tested negative) felt supported in making a decision that aligned with her values: “This geneticist understood my reasoning and whether she agreed with it or disagreed, she wasn't judgemental, and just supported me”.

Tori (20–29, tested positive) felt she was discouraged from having the test and needed to persuade the genetics team she was ready to make the decision: “I felt like I was getting nowhere, because it was just going round in circles, it was just like, ‘You're too young, you don't need to worry about it now’, and it's like, ‘But that's my decision, not yours’… I did feed back about how I felt… I said, ‘But in my case, you've not protected me, you've actually made this a really stressful experience because I just want you to help me and I've told you what I want, and stop being a blocker’.”

Ian (50–59, tested positive) felt the healthcare team didn't want him to have testing: “They just said that they didn't want me to take the test. They said that you are better off not knowing. And telling me a statistic of something like there's only sixteen percent of people want to know.”

### 3.4 Making sense of ALS and being at risk

Participants had varied knowledge of ALS at the time of seeking genetic counseling, and wanted more details about the disease including: causes and triggers of ALS, including evidence on delaying onset; ALS symptoms and progression; coping and living with ALS; treatment, support and management; and long‐term planning such as financial planning and end of life care. However, some found the information they were given on the trajectory of living with ALS to be distressing at the time of making genetic testing decisions:

I felt like it was just all about, this is what's going to happen… if you get it, depending on which one, how long you've got to live. It just felt like more of the negative side of it. (Tori, 20–29, tested positive)

She did mention something about not being able to swallow, one of the first signs. So then you just think about it all the time, don't you? (Ian, 50–59, tested positive)

Knowledge of the disease gained from experiences with relatives could impact people at the point of decision‐making. Angus (30–39, waiting for results) was seeing his father's condition decline and felt “clouded by emotional judgement”: “That was my biggest concern about making the decision to have it [predictive testing], was that my brain would go into overdrive with worrying about what might happen.” Angus and Toby felt increased awareness on cognitive and behavioral changes associated with ALS‐linked genes could help people better understand their family members' symptoms and reduce stigma.

Finding out about the genetic overlap between ALS and FTD seemed to result in confusion at times, especially where this did not align with the individual's family history: “The shock value of it [the gene variant in the family] being linked to MND was quite profound… I'd never known or heard of that possibility before, so suddenly to have a fact presented to you at that stage was almost alien to my thought channels and I don't think I could process the information correctly” (Evelyn, 60–69, tested negative).

Information gained indirectly from previous consultations, such as when a relative was living with ALS, at times impacted on people's understanding and reasoning about the decision. This was especially the case where there had been no opportunity to ask follow up questions at the time and they'd not seen a genetic counselor themselves since: “I find it confusing… they did just casually throw in that average age of onset for familial motor neuron disease is age 51, and when I was in my 40's, it became very much in my mind as I approached 51… But now I find it reassuring… I wish it had been explained to me more and we'd been given more chance to ask questions. What happens if you live to be 60? What happens if you live to be 65, what are the odds of getting it then?… that would help me decide on whether I was going to get tested.” (Laurence, 50–59, not tested).

Participants tended not to have in‐depth knowledge about genetics and the complex genetic mechanisms associated with ALS. Ben (40–49, not tested) did not think knowing more about genetics and ALS would affect his decision or be relevant to him: “It's a binary question at the end of the day, will I get it or won't I? And knowing any more detail than that… is just information.”

Generally, though, participants felt basic resources on genetics, and gene‐specific information on inheritance, penetrance, age of onset, and the link between particular genotypes/ phenotypes would be useful for decision‐making about testing, and future planning. Facts about genes and transmission are core to genetic counseling consultations. However, there was evidence of variation in experiences of how these facts were discussed, and understood, within predictive testing consultations:

“I said to her [genetic counsellor], ‘So what are the chances?’ And she was like, ‘Well, pessimistically, probably a 100% chance that you'll have the gene, given your family history, but optimistically we still say that there's a good chance that you won't present’” (Angus, 30–39, waiting for results). Angus recalled being told that if he did carry the *C9orf72* gene, it would be 50/50 whether it would manifest as ALS or FTD.

Interviews suggest participants were accepting of uncertainty and felt the limitations of testing should be communicated to those contemplating the decision:

Obviously there wasn't anything that concrete about it but actually just to know that some genes have got a really high penetration, some genes don't, all that sort of thing was quite useful for me. (Martina, 40–49, tested positive)

It's being able to say to people, ‘… depending on which gene you're diagnosed with, we really don't know a lot about it, and we might not be able to counsel you any better than whether you knew or not’…I don't ever look back and think, ‘I wish I'd have known that, it would have changed my mind’, but it is something that if people are really swaying it might sway them one way or another. (Julia, 30–39, tested positive)

Participants' understanding of test results suggests there can be confusion about what results mean. Findings raise the question of whether information givers may at times simplify explanations to a binary yes (of getting ALS) or no (of not getting ALS):

Craig (50–59, tested positive) recalled being told at his results consultation that he would “most likely go on to develop Motor Neuron Disease and/or early onset dementia,” and asked for clarification on what this meant: “I said something to him like, ‘What do you mean by most likely?’ and he said, ‘Well, yes, you will’”.

Ian (50–59, tested positive) remained unsure of his risk of ALS or FTD even after genetic counseling and his own positive result: “I'm not sure if I've got the one for is it full frontal dementia, or MND. I am just not sure. Is it the same? Can it go one way or the other, or are you guaranteed to get one?”

Uncertainty could also continue after a negative result; Evelyn (60–69, tested negative) recalled her results consultation: “[Genetics consultant] said to me ‘look, it's very clear you don't have this gene, but we don't know about any others and it doesn't mean you won't get some form of dementia’… I didn't ask anything further because as I say I was so shocked, stunned in fact by the result, that I was just trying to digest what I'd been told.” She described this ongoing risk as a “sword of Damocles” over her head.

### 3.5 Satisfaction with genetic counseling and information provision

Participants expressed varying levels of satisfaction with the genetic counseling process, reflecting differences in their personal situations, professionals' approaches to communication, and the complexity of decision‐making.

Genetic counseling was seen as an essential part of the testing process and interviews demonstrated good practices around personalization of information and providing the right information at the right time.

Megan (30–39, tested positive) recalled: “I think the genetic counsellor that I had pitched it really well because she knew that I was a [healthcare professional] so she didn't give me a whole load of unnecessary information…she obviously checked my understanding but then kind of left me to it.”

Evelyn (60–69, tested negative) felt the pacing of information across consultations helped with making sense of the decision and testing process: “She [genetic counsellor] knew at that stage it was very early days, so it was pointless again going into too much detail because we didn't know the outcome and she didn't want to frighten me to death with facts and figures, but by the same token she wanted me to understand the importance of the decision I was making.”

Participants who had discussed genetic counseling experiences with family members perceived there to be variations in practice, with some genetic counseling focusing on risks and poor outcomes, and some on advances in research and care.

Tori (20–29, tested positive) found the focus on how she might feel if she tested positive unhelpful: “I felt like when I had the genetic testing, it was very doom‐and‐gloom… 'You're going to get it, it's going to kill you, we just don't know when' …And I felt like what my Auntie got was a lot more positives… the trials and things that were going on, and the positive space and what the charity was doing”.

Some participants felt the information provided didn't support their decision‐making, wanting different types of information and support relevant to their situations. Megan (30–39, tested positive) missed having a comprehensive overview: “I got snippets of information from here and there and I put them all together, but I don't remember it ever being set out for me.”

Ian (50–59, tested positive) felt the level of detail was too complex to support decision‐making: “She's good at what she does but she's putting 2,000,000% too much information into her conversations, and I didn't want to tell her, ‘Whoa, whoa, whoa’. If you are explaining something, do it in layman's terms.”

Craig (50–59, tested positive) expected more emotional support, particularly at the point of being given his result: “He [genetic counsellor] didn't really offer anything, he seemed quite tongue‐tied…I basically was like, ‘Right, well is that it then?’ and this other guy [genetics consultant] said, ‘Well yes, basically’ and I just had to get up and leave, and I felt it could have been much more user‐friendly. If that guy was a counsellor, he could have actually been there to talk to me about it.”

Angus (30–39, waiting for results) wondered if there was another forum to talk about the emotional consequences of predictive testing, such as peer‐led support: “Is there a system in place that I can contact someone and have a chat about decision‐making?… so that it's not something that is made clinical and sterile.”

Laurence felt being able to get in touch with healthcare professionals to ask questions was essential after a consultation, as it can take time to digest this emotional and complex information.

Overall, participants expressed a desire for clear, accessible information resources, pitched at an appropriate level, in a range of formats, tailored to individual circumstances, and addressing the emotional impact of the testing decision and results.

## 4 DISCUSSION

Predictive genetic testing is a complicated decision, requiring individuals to navigate complex and uncertain information, a rapidly progressive research landscape, and a range of personal and family considerations. Knowing how best to support people in making informed decisions about having testing or not is challenging for many professionals working with families affected by ALS. In this context we sought to understand people's experiences of making decisions around predictive testing and of the genetic counseling and predictive testing pathway.

A central tenet of genetic counseling is respect for autonomy, yet some participants felt they were discouraged from having predictive testing. Perceiving directive advice in this scenario may be detrimental to informed decision‐making, where people should be supported to make reasoned choices based on their own values and priorities. Indeed, participants who felt their genetic counselor understood, listened to, and supported them without judgment generally expressed a more positive experience than those who felt this was not the case. This reflects wider research which highlights that people report higher satisfaction in genetic counseling when the relationship is characterized by empathy (Jamal et al., 2020) and less where the healthcare provider is seen as cold or emotionally distant (Guimarães et al., 2013).

Less positive experiences were reported by those who felt they had to convince their genetic counselor of their reasoning and overcome barriers, which could lead to frustration and in one case a request for no further contact following results. People at risk of other late‐onset neurological conditions have also described a sense of “going through the motions” to access predictive testing (Forrest Keenan et al., 2015); in these cases, the genetic counselor becomes seen as a “gate keeper” (Guimarães et al., 2013). As in our study, where genetic counseling became a “battle” to seek the test, this could negatively impact relationships and cause distress (Forrest Keenan et al., 2015). Further, participants at times appeared to lack clarity around the role of the genetic counselor; some struggled to untangle genetic counseling from therapeutic counseling, and perceived it as unsatisfactory if their expectations were not met.

It is worth noting that participants often made decisions about predictive testing outside of the pathway of care, before seeking support from their clinical service. These participants described being sure of their decision, seeking genetic counseling to facilitate having the test rather than to support decision‐making, a point which has been noted in previous research on other genetic conditions (MacLeod et al., 2014). These different goals may impact people's views on and satisfaction with the genetic counseling process; having multiple consultations with a time gap in between was frustrating for participants who felt sure of their decision and satisfied with their understanding, or who had pursued the test at a point where it fit with other events and responsibilities in their life (e.g., work and family considerations). Research on other late‐onset neurological conditions has reported the structure of genetic counseling provides a supportive space for some, but may also be seen as repetitive, inflexible, difficult, and lengthy, especially for those who are certain they want the test from their first consultation (Forrest Keenan et al., 2015; Guimarães et al., 2013). As such, what is seen as “good practice” is not necessarily aligned with participant's preferences, where what is perceived as supportive may be based on their stage of decision‐making and personal context.

Information is impactful. This study has shown how some perceived information as beneficial in reducing uncertainty and supporting decision‐making, but also how it could cause overwhelm, confusion, and anxiety. People engaging with predictive testing decisions come to genetic counseling with varying levels of existing knowledge and a variety of approaches to how much they want to know. Participants appreciated a personalized approach to information provision, reflecting wider research where participants valued receiving information adjusted to their existing knowledge and presented in a different way to what they had previously been told or read (Guimarães et al., 2013). There were some, however, who struggled to find comprehensive, accurate, and accessible information that met their needs. Further, being told detailed descriptions of ALS symptoms and living with a worsening condition was seen as unhelpful for some when making the testing decision. This included one person who found the tone of the counseling focused too much on negative aspects, which impacted rapport, suggesting information on areas such as research progress and participation opportunities may provide balance and be well received.

A defining feature of ALS predictive testing is uncertainty, including around the implications of a positive result (Dratch et al., 2023). However, our findings suggest such uncertainties are not always fully explained or understood. Participants expressed challenges in making sense of genetic concepts and their personal risk, reflecting recent research with a similar population (Howard, Mazanderani, et al., 2024). Participants were left with partial understandings and unanswered questions on aspects including penetrance, the genetic link between ALS and FTD, and the possible implications of predictive test results even after genetic counseling. It is not possible to say whether these views represent a misinterpretation of facts, poorly explained information, or a misremembering of information over time. Regardless of how participants came to these views, or what information was provided by genetic counselors and other healthcare professionals, it is people's understanding itself that is significant here. The findings raise doubts over whether existing verbal information provision alone is sufficient to support people making truly informed decisions about predictive testing.

Decision‐making around predictive testing takes place in family and social contexts (Mendes et al., 2011). Decisions about testing are often considered and carried out in a period of stress, alongside the diagnosis and illness of family members, grief and bereavement, other relatives' predictive testing, and life events such as reproduction (Crook et al., 2022). Research among people seeking predictive testing for HD has found a perception that pre‐test counseling did not address the emotional aspects of living with risk and experiences of the disease in the family (Forrest Keenan et al., 2015). Our research has picked up on the need for genetic counseling that is responsive to the different stage‐of‐life concerns of people making decisions about testing. Further, decision‐making may be underpinned by dilemmas around family communication and disclosure to children (Mendes et al., 2011), an area where additional support may be needed (Crook et al., 2022). In other genetic contexts, healthcare‐mediated disclosure options, particularly family letters, have been identified as supportive when used in conjunction with family‐mediated disclosure (Öfverholm et al., 2024). This option is one among several which could be explored in future research in ALS.

This study provides evidence of what is important to people making decisions around ALS predictive testing, and suggests there is a role for patient decision aids which people can access independently, alongside the support of clinical services. We used these findings, and those from our studies with healthcare professionals and people living with ALS (Howard, Bekker, et al., 2024, 2023, 2025), to inform the content of two patient decisions aids (“Genetic testing and MND,” and “Predictive genetic testing and MND”) (Howard, McNeill et al., 2025a, 2025b). These publicly available patient decision aids are judged independently as meeting the criteria needed to proactively support people with ALS and their family members to make informed decisions between testing options (OHRI, 2025). The next phase of our research is to explore integration and implementation of the decision aids within relevant settings and services. Findings from this research may also have relevance for genetics professionals working in ALS predictive testing (summarized in Table 2).

**TABLE 2:** Implications of findings for predictive testing and genetic counseling practice.

Challenge/need identified	Implications and suggestions for practice
Participants sometimes struggled to understand the role of the genetic counselor (as distinct from therapeutic counseling) and the protocol for predictive testing in amyotrophic lateral sclerosis (ALS).	Explaining the role of genetic counselors and process of genetic counseling can ensure expectations are aligned. Understanding the rationale for this process may be useful for people attending genetic counseling.
Participants had varied information needs and approaches to how much information they wanted to know about ALS, genetics, and genetic risk. Participants appreciated tailored information provision that responded to their situation and sometimes felt this was not provided.	People may benefit from genetic counseling that assesses their existing knowledge and information needs to inform information provision, particularly in relation to genetics and inheritance, and the meaning and implications of results. It can be useful to explore each person's preferences to find the balance between making sure they are informed and ensuring they don't feel overwhelmed by the amount, complexity, or tone of information given.
People's understanding of information may be influenced by many factors. Some participants struggled to understand or could not recall key information related to ALS, implications of testing, and their results. Understandings may change over time.	Exploring how people understand and recall information in subsequent appointments can ensure key information has been clearly communicated in an accessible way. People may appreciate written information and resources to return to over time to consolidate knowledge, support decision‐making, and family information sharing. People should know where to find information and support.
A positive predictive test leaves uncertainties over if, when, and how symptoms may manifest. Participants found uncertainties challenging to make sense of when expressing the meaning of results. From this study it is not possible to say how uncertainties are communicated in genetic counseling, but findings suggest people have unanswered questions.	People may benefit from clear communication on the uncertainty of predictive testing, particularly in relation to gene‐specific penetrance, phenotypic variability of ALS‐linked genes, and age of onset. This should happen before testing and be returned to over the genetic counseling process, including when explaining results.
Decisions around predictive testing are often intertwined with or lead to other decisions, including around family planning (and reproductive genetic testing options) and research participation. People may be considering their predictive test decision in this context.	People may wish to receive information and support on related/ intertwined decisions, as appropriate.
Managing information sharing and disclosure to relatives, including children, can be challenging for some. In some cases, this fed into decision‐making around predictive testing, caused worry for the future, or led to (plans for) partial or non‐disclosure of information that may have relevance for family members.	Exploring dynamics around family communication, and plans for future conversations, may be a useful starting point for providing support as needed.
Having ALS in the family and living with risk may raise challenges and emotions related to memories of the disease in family members; current experiences of the disease and living with risk; and uncertainty, anxiety, or dread over future illness (for the individual and/ or the wider family). People will have varied experiences and responses to their situation.	These personal and family experiences are important to explore in genetic counseling as they may inform each person's perspectives and decision‐making.
Receiving predictive test results is often an emotional and challenging time. Some participants struggled to find support and did not feel their needs were met. This appeared to heighten distress and caused dissatisfaction with the predictive testing experience.	People should be made aware of what post‐test support is available (including lack of support) before having predictive testing. More detail can be given after results, but this information may be relevant for decision‐making.

A limitation of this study is that the majority of participants were White British. Understanding how socio‐cultural factors may impact experiences and engagement with services should be explored in future work and used to inform decision support tools to ensure these are widely accessible and culturally sensitive. Further, whilst multiple participants had not been tested, including more people in this study who had genetic counseling and decided not to proceed with the test at present could have elucidated novel insights. Alongside individual interviews, methods such as recording clinic consultations of people making testing decisions could support deeper understanding of genetic counselors' communication and the “processes of translation and interpretation” (Atkinson et al., 2013) that are applied to genetic information in the context of making choices on predictive testing.

The context of this study (informing the development of a patient decision aid) shaped this research, including the sampling strategy. When designing decision aids, there is a need to understand the trade‐offs people are making when deciding between having and not having a test, and their information and support needs over this process. This reasoning may change over time, hence the decision to include participants with varied testing decisions and results, at different points in the testing pathway (including, for a small number of participants, before formal engagement with genetic counseling services, as decision aids will be available publicly and may be used by people who have not sought genetic counseling). This breadth meant numbers of each group were small, yet the authors perceived the data sufficient to provide rich information and support the goal of the study (Malterud et al., 2016). Focused studies on particular timepoints, for example in the period following delivery of test results, are important to inform future interventions and meet people's needs across their predictive testing journey.

## 5 CONCLUSION

This study contributes an important understanding of participants' decision‐making and support needs around ALS predictive testing, both as they engage with the decision and go through the genetic counseling and testing process, and as they reflect back on their experiences. This is part of a larger, mixed methods study, carried out to inform the development of patient decision aids to support people with ALS and their family members to make informed choices around ALS genetic testing and predictive testing. The need for decision support is reinforced in the above findings, given inconsistencies of genetic counseling practice, variation in information provision, and unmet information needs. We hope the decision aids will provide a tool to support people to make informed and reasoned choices that are right for them, to be used alongside important clinical expertise and guidance. This is a timely area given the likely increase in interest around predictive testing and demand on genetic counseling services.

## AUTHOR CONTRIBUTIONS

**Jade Howard:** Conceptualization; formal analysis; investigation; project administration; writing – original draft; writing – review and editing. **Hilary L. Bekker:** Conceptualization; funding acquisition; writing – review and editing. **Christopher J. McDermott:** Conceptualization; funding acquisition; writing – review and editing. **Alisdair McNeill:** Conceptualization; funding acquisition; writing – review and editing.

## CONFLICT OF INTEREST STATEMENT

The authors report no competing interests.

## ETHICS STATEMENT

Ethics approval for this study was granted by a UK NHS Research Ethics Committee (22/SW/0047) and the University of Sheffield (050846). All applicable international, national, and/or institutional guidelines were followed.

## PATIENT CONSENT STATEMENT

Participants consented to take part in the interview and gave consent for their material to be used in publications.

## Supporting information


Data S1.

## Data Availability

Research data are not shared.
