# Supplementary material for: Predictive genetic testing in amyotrophic lateral sclerosis (ALS): Experiences of decision‐making and engagement with UK genetic counseling services
Source: J Genet Couns. 2026 Feb 19;35(1):e70184. doi: 10.1002/jgc4.70184 (PMC12920051; doi:10.1002/jgc4.70184)
Supplement: Supplementary file 1 — Data S1. [file JGC4-35-0-s001.docx]

***Supplemental materials***

**Topic guide – predictive testing interviews**

**Experiences of (inherited) MND in the family**

- Would you like to start by telling me about your experiences of MND (in the family)?
- What have you been told about genetics in MND/ the possibility that MND can be passed on in families?
- How have you discussed inherited MND with your family members*?*

**Genetic testing of a family member with MND**

- Can you tell me about your experiences of your family member’s genetic testing?
- How was finding out this information for you?

**Predictive genetic testing**

- What have you been told about predictive testing options?
- Can you tell me about your experiences of predictive testing?
- How did you come to a decision?
- What factors were involved?

**Information and support around genetic testing**

- What information would it be helpful for families to get (from a clinician) when considering genetic testing?
- What support do you think people should be offered at the time of/ after predictive testing?
- Is there anything you’ve found hard to understand or would like to have been explained better?
- Is there any other information or support you’d like to be given?
